# Distribution and Ecophysiology of Calanoid Copepods in Relation to the Oxygen Minimum Zone in the Eastern Tropical Atlantic

**DOI:** 10.1371/journal.pone.0077590

**Published:** 2013-11-05

**Authors:** Lena Teuber, Anna Schukat, Wilhelm Hagen, Holger Auel

**Affiliations:** Bremen Marine Ecology, University of Bremen, Bremen, Germany; Institute of Marine Research, Norway

## Abstract

Oxygen minimum zones (OMZs) affect distribution patterns, community structure and metabolic processes of marine organisms. Due to the prominent role of zooplankton, especially copepods, in the marine carbon cycle and the predicted intensification and expansion of OMZs, it is essential to understand the effects of hypoxia on zooplankton distribution and ecophysiology. For this study, calanoid copepods were sampled from different depths (0–1800 m) at eight stations in the eastern tropical Atlantic (3°47′N to 18°S) during three expeditions in 2010 and 2011. Their horizontal and vertical distribution was determined and related to the extent and intensity of the OMZ, which increased from north to south with minimum O_2_ concentrations (12.7 µmol kg^−1^) in the southern Angola Gyre. Calanoid copepod abundance was highest in the northeastern Angola Basin and decreased towards equatorial regions as well as with increasing depth. Maximum copepod biodiversity was observed in the deep waters of the central Angola Basin. Respiration rates and enzyme activities were measured to reveal species-specific physiological adaptations. Enzyme activities of the electron transport system (ETS) and lactate dehydrogenase (LDH) served as proxies for aerobic and anaerobic metabolic activity, respectively. Mass-specific respiration rates and ETS activities decreased with depth of occurrence, consistent with vertical changes in copepod body mass and ambient temperature. Copepods of the families Eucalanidae and Metridinidae dominated within the OMZ. Several of these species showed adaptive characteristics such as lower metabolic rates, additional anaerobic activity and diel vertical migration that enable them to successfully inhabit hypoxic zones.

## Introduction

The vertical and horizontal expansion of oxygen minimum zones in tropical oceans is a crucial factor for marine organisms influencing their distribution ranges, behavioural patterns and metabolic rates [1]–[3]. Oxygen depletion has been identified as one of the major future hazards to marine ecosystems and global fisheries [2], [3]. Since the 1960s, the tropical Atlantic has suffered from the most severe decline in oxygen concentration of ∼0.5 µmol kg^−1^ year^−1^ and the area affected by oxygen minimum zones (OMZs) has expanded in spatial extent by 4.5 million km^2^ [3]–[5]. Recently, minimum oxygen concentrations of 17 µmol kg^−1^ have been measured at intermediate depths in the eastern tropical Atlantic [6], [7]. Nevertheless, the OMZ in the Atlantic is not as pronounced as in the eastern tropical Pacific and Arabian Sea, where oxygen concentrations are often below 4.5 µmol kg^−1^ [7], [8].

Expanding OMZs are a severe problem restricting the horizontal and vertical distribution as well as migrations of marine organisms [9]–[12]. Zooplankton organisms are especially susceptible and sensitive to changing environmental factors [13] and zooplankton biomass is usually severely reduced within the centre of the OMZ [10]. In contrast, gradients at the upper and lower margin of the OMZ are often hotspots of biological activity [9], . Interestingly, certain copepod and euphausiid species are able to live within or migrate through the OMZ [15]–[17] and the copepods *Calanoides carinatus*, *Rhincalanus nasutus* and *Pleuromamma robusta* frequently inhabit the OMZ in the Atlantic [11], [18]. Vertical migration through the OMZ is apparently associated with a reduction of respiration rates at lower oxygen partial pressures [19] and a higher activity of the enzyme lactate dehydrogenase (LDH), which indicates anaerobic metabolism [20], [21]. Zooplankton mainly survives aerobically within OMZs [22], but the anaerobic pathway may serve as an additional energy supply to support activity above routine metabolism [21], [23].

The influence of low ambient oxygen concentrations on zooplankton metabolism has been investigated in the OMZs of the major eastern boundary upwelling systems off Peru, California and Namibia [11], [23], [24]. During hypoxia tolerance measurements, *C. carinatus,* the dominant copepod species in the Benguela upwelling system, survived surprisingly low oxygen concentrations of ≤1 ml l^−1^, but could not tolerate hypoxic conditions below 0.8 ml l^−1^ in the centre of the OMZ [11]. Apart from changes in body mass and temperature, the copepods *R. nasutus* and *Metridia lucens* from the OMZ off Namibia showed a reduction in respiration rate by 62% and 43%, respectively, as compared to specimens from the surface [25]. Besides metabolic suppression, an efficient removal of oxygen from the water or an additional energy supply via anaerobic metabolism allow pelagic crustaceans to survive in hypoxic regions [22]. Zooplankton organisms specifically adapted to hypoxic conditions may thus find refuge within the OMZ from predation and competition by less tolerant species [11], [26], [27].

Zooplankton studies in the tropical and subtropical Atlantic have often focused on the abundance and distribution of copepods [28], [29]. There is an increasing number of studies on influences of hypoxia on marine species, but ecophysiological data of copepods from the tropical Atlantic, investigating the effects of OMZs on distribution patterns and metabolic rates, are still limited. It is essential to understand the physiological capacities of different copepod species to assess potential implications of expanding OMZs on zooplankton communities. The aim of this study is to analyse the impact of the OMZ in the eastern tropical Atlantic on the distribution and metabolic activity of common calanoid copepods. This paper fills a gap in the limited data set on the ecophysiology of tropical Atlantic copepods and contributes to a better understanding of the zooplankton community structure and adaptive processes in the light of predicted expanding OMZs in the future.

## Materials and Methods

### Ethics Statement

The present study on planktonic copepods does not include protected or endangered species. No specific permissions were required for sampling in the open tropical Atlantic Ocean, since sampled stations were positioned in international waters.

### Sampling

**Figure 1:**
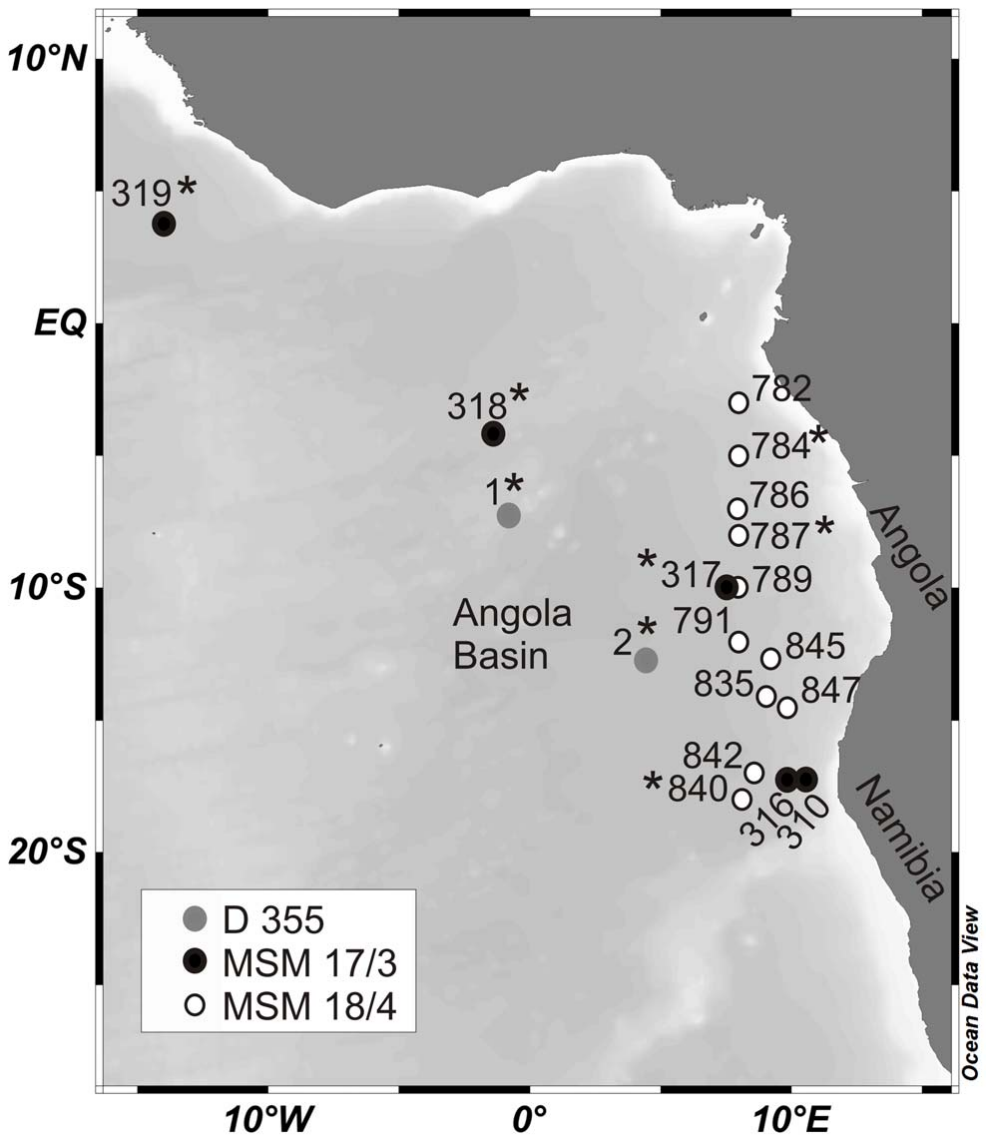
Sampling stations in the eastern tropical Atlantic Ocean. Copepods were sampled during three expeditions with RRS *Discovery* in 2010 (D355, gray circles) and with RV *Maria S. Merian* in 2011 (MSM 17/3, black circles and MSM 18/4, white circles). * = stations analysed for copepod abundance and species composition.

Mesozooplankton was sampled in the eastern tropical Atlantic Ocean during three expeditions in September 2010 (RRS *Discovery*, D355), February/March 2011 (RV *Maria S. Merian*, MSM 17/3) and July/August 2011 (RV *Maria S. Merian*, MSM 18/4) (Table 1, Figure 1). Stratified vertical hauls were collected with a Multinet Midi equipped with two flow meters (HydroBios, Kiel, Germany; mouth opening: 0.25 m^2^, mesh size: 300 µm). Some specimens were also collected from a double (18 nets) and single (9 nets) MOCNESS (Multiple opening/closing net and environmental sensing system, mouth opening: 1 m^2^, mesh size: 333 µm, [30]). At each station, five discrete depth layers were sampled by Multinet selected according to the local hydrographical regime (temperature and oxygen) determined by CTD casts (Table 1). In particular, the vertical extent of the oxygen minimum zone (OMZ) was reflected in the depth intervals with discrete samples collected above, within and below the OMZ. The volume of water filtered by the Multinet was determined by calibrated flow meters and was usually considered 25 m^3^ per 100 m depth interval and, thus, ranged from 7.5 m^3^ for a 30 m depth interval at the surface to 200 m^3^ for the deepest depth interval from 1800 to 1000 m during expedition MSM 18/4 (Table 1). Vertical profiles of oxygen concentration, temperature and fluorescence were measured by corresponding CTD casts at each station, except for stations 1 and 2 in 2010. Water layers with ≤45 µmol kg^−1^ of dissolved oxygen were considered as OMZ according to [7].

**Table 1:** Sampling data.

Cruise	Station	Date (D/M/Y)	Time gear atdepth [UTC]	Positionlatitude	Positionlongitude	Sampling intervals [m]	O_2_ min[µmol O_2_ kg^−1^]	O_2_ mindepth [m]	SST [°C]
**D 355**	1*	01.09.2010	15∶43	7°15′S	0°45′W	800-500-200-100-50-0	n.d.	n.d.	n.d.
	2*	03.09.2010	14∶30	12°42′S	4°26′E	800-500-220-120-80-0	n.d.	n.d.	n.d.
**MSM 17/3**	310	22.02.2011	17∶07	17°15′S	10°30′E	1000-**500-200-100**-50-0	14.6	258	24.8
	316	24.02.2011	04∶01	17°15′S	10°00′E	1000-**500-200-100**-50-0	15.1	363	24.4
	317*	25.02.2011	22∶50	10°00′S	8°00′E	1000-**600-300**-100-50-0	17.6	427	28.9
	318*	28.02.2011	00∶30	4°07′S	1°26′W	1000-**550-350**-100-50-0	42.6	417	28.4
	319*	04.03.2011	04∶12	3°47′N	13°58′W	1000-400-200-100-50-0	56.1	283	29.6
**MSM 18/4**	782	25.07.2011	15∶52	3°00′S	8°00′E	1800-1000**-**500-150-30-0	50.0	204	22.1
	784*	26.07.2011	06∶43	5°00′S	8°00′E	1800-1000-**600-150**-30-0	39.8	248	24.1
	786	26.07.2011	21∶54	7°00′S	8°00′E	1800-1000-**400-250**-30-0	21.5	336	23.1
	787*	27.07.2011	06∶32	8°00′S	8°00′E	1800-1000-**450-200**-30-0	18.6	336	22.9
	789	27.07.2011	21∶48	10°00′S	8°03′E	1800-1000-**600-250**-40-0	16.7	397	22.2
	791	28.07.2011	12∶32	12°00′S	8°00′E	1800-1000-**600-250-50**-0	18.7	334	20.7
	835	05.08.2011	17∶49	14°00′S	9°06′E	1800-1000-**450-200-50**-0	17.4	378	19.5
	840*	07.08.2011	06∶08	18°00′S	8°00′E	1800-1000-**450-250**-85-0	16.4	324	17.9
	842	07.08.2011	16∶42	17°00′S	8°34′E	1800-1000-**400-250-85**-0	22.6	331	17.8
	845	10.08.2011	23∶08	13°00′S	9°05′E	1800-1000-**600-200**-30-0	12.7	410	20.5
	847	12.08.2011	21∶36	14°30′S	9°51′E	800-**500**-**300-150-50**-0	17.9	341	18.7

Sampling intervals highlighted in bold numbers indicate the approximate vertical extent of the oxygen minimum zone (O_2_≤45 µmol kg^−1^). D = *Discovery* cruise, MSM = *Maria S. Merian* cruises, UTC = universal time code, O_2_ min = lowest oxygen concentration at the respective station, O_2_ min depth = depth of the oxygen minimum at the respective station, SST = sea surface temperature, n.d. = no data, * = stations analysed for copepod abundance.

Mesozooplankton samples were sorted under a dissecting microscope, and only calanoid copepods in apparently good condition were used for respiration experiments on board. Copepods were staged and identified according to [31]. Additional copepod specimens were deep-frozen at −80°C for later enzyme activity analyses of the electron transport system (ETS) and lactate dehydrogenase (LDH). The remains of the samples were preserved in a 4% buffered formaldehyde in seawater solution for the analysis of copepod abundance, vertical distribution and species composition.

### Copepod Abundance and Data Analysis

Formaldehyde-fixed zooplankton samples of eight stations from three cruises (D 355: stns. 1 and 2 (day); MSM 17/3: stns. 317, 318, 319 (night); MSM 18/4: stns. 784, 787, 840 (dawn); Figure 1, Table 1) were chosen for the analysis of abundance and vertical distribution of calanoid copepods. The analysis focused on abundant and larger calanoid species that regularly occurred in the samples and were also used for respiration measurements and biochemical analyses. These copepods were counted from the entire sample and all specimens were staged and identified under a dissecting microscope to species or genus level according to [31]. The counted number of individuals per taxonomic category and sample ranged from a few individuals in rare deep-sea species to over 150 individuals per sample in abundant epipelagic taxa. For species that occurred only sporadically as single individuals in the samples, abundance values were not calculated. Instead, these species were listed as “single individuals” (s.i.). Specimens used for respiration measurements and/or biochemical analyses were added to the total number for the calculation of abundance.

**Figure 2:**
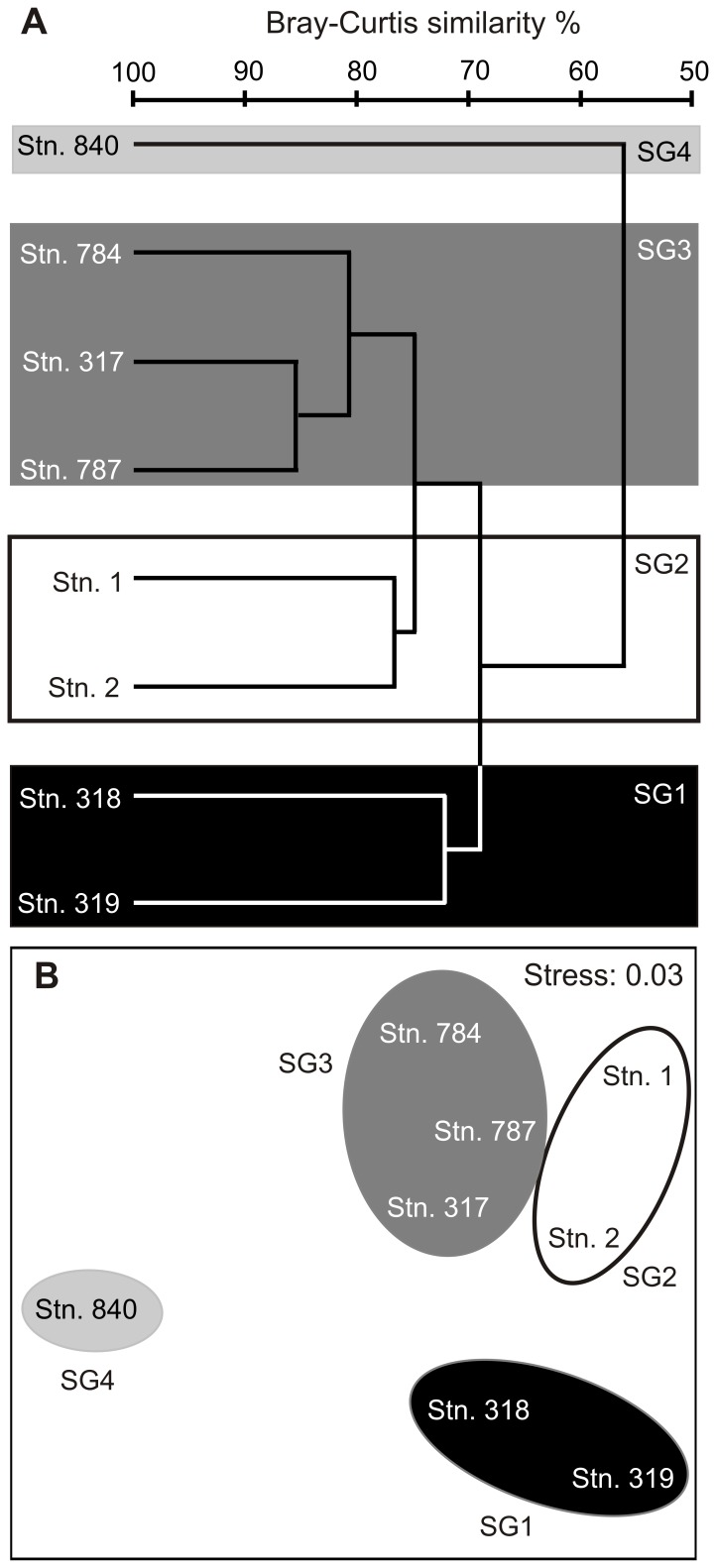
Cluster of stations with similar faunistic composition and derived station groups (SG1-4). A, dendrogram. B, multidimensional scaling (MDS) plot.

To identify different biogeographical regions characterized by distinct copepod communities, a cluster analysis was performed with PRIMER v6 software [32] based on a species-station table compiling copepod abundance (no. of ind. 1000^−1^ m^−2^) in the upper 1000 m water column. Abundance data were fourth-root-transformed to minimize the dominance of highly abundant species and to increase the relevant impact of rare ones. Pair-wise similarity between each pair of stations was calculated by the Bray-Curtis similarity index based on the species-station table. For the cluster analysis a group average technique was applied. Results of the cluster analysis are presented in a dendrogram and a multidimensional scaling (MDS) plot (Figure 2). Based on the results of the cluster analysis, four station groups (SG1 to SG4) were derived and compared in terms of copepod abundance and distribution. Abundance data for each station will be made available via the PANGAEA database.

### Respiration Measurements

Respiration measurements were performed on board of all three research vessels by optode respirometry with either three 1-channel or one 10-channel optode respirometer (Fibox 3 and Oxy-10 Mini, PreSens Precision Sensing GmbH, Regensburg, Germany). Oxygen concentration was measured via oxygen minisensors (ø 5 mm) attached to the inner wall of gas-tight Winkler bottles (12–13 ml volume). Incubation bottles were filled with oxygenated seawater previously filtered (0.2 µm, Whatman GF/F filter) and UV-sterilised by slowly flowing through the container of an ultraviolet lamp (Aqua Cristal UV C5 W, JBL) to reduce microbial respiration. All specimens were acclimated at experimental temperature for several hours prior to the experiments and were not fed before and during experiments. Depending on the body size of each species, incubation bottles contained 1 to 10 individuals. Only specimens in apparently good condition were chosen for experiments. All experiments were run in darkness in water baths placed into temperature-controlled incubators to ensure constant temperatures throughout the experiments. Incubators were set to different target temperatures (5–20°C) according to *in situ* conditions at the corresponding sampling depths, tolerating a deviation of ±1°C. Measurements lasted for at least 6 to 8 h. For each experimental setup, one to two animal-free controls were run under the same conditions to compensate for potential microbial respiration. After the experiments, all specimens were deep-frozen at −80°C for later dry-mass determination after lyophilisation for 48 h. Intra-specific differences in respiration between adults and copepodite stages as well as depth-related differences in respiration were statistically evaluated by Mann-Whitney U tests and Kruskall-Wallis tests, followed by a Dunn’s multiple comparison test, respectively [33].

### Enzyme Activities

Electron transport system (ETS) activity was measured according to standard methods [34], [35] and optimized for copepod species [36]. The phosphate buffer (PHB, 0.1 M, pH 8.0) contained Triton X-100 (0.2% v/v), while the amount of polyvinylpyrrolidone (PVP) was reduced to 0.5 mg ml^−1^ in the homogenizing buffer (HOM, 75 µM). For the substrate solution (SUB), NADH (1.3 mM), NADPH (0.05 mM) and succinate (1 mM) were dissolved in PHB. The 2-(p-iodophenyl)-3-(p-nitrophenyl)-5-phenyl tetrazolium chloride (INT) solution (2.5 mM, pH 7.5) was dissolved in de-ionized water. Wet mass (WM) of frozen copepods was determined and specimens were immediately homogenized for 2 min in a reaction cup (2 ml, Eppendorf safe-lock tube) using a hand pistil. The homogenate contained 1 mg copepod per ml HOM. Homogenates were centrifuged at 4700 g for 10 min at 0–4°C. All steps were conducted on ice. The final reaction mixture was reduced to 1 ml, containing 600 µl SUB, 200 µl INT and 200 µl supernatant. After a species-specific incubation time (10 to 60 min), absorbance was measured in 2 ml quartz cuvettes under non-limiting substrate conditions in a temperature-controlled photometer (490 nm wavelengths, Kontron Instruments, UVIKON 941) at *in situ* temperature and with distilled water as reference. No quench solution was used. For each sample, four replicates were measured as well as three sample blanks (600 µl PHB, 200 µl INT, 200 µl supernatant) and three substrate blanks (600 µl SUB, 200 µl INT, 200 µl HOM).

Lactate dehydrogenase (LDH) activity was measured according to standard methods [20],[37] and optimized for copepod species. LDH assays were performed on a mixture of Tris/HCl buffer (80 mM, pH 7.2 at 20°C), pyruvate (2 mM), NADH (150 µM) and KCl (100 mM). The initial preparation of the homogenate was similar to the ETS activity assay, except that the homogenization dilution was usually 1 mg copepod per 24 µl homogenizing buffer (Tris HCl buffer, 0.01 M, pH 7.5 at 10°C). Homogenates were centrifuged at 6600 g for 10 min at 0–4°C. All steps were conducted on ice. The total volume of the assay medium in the cuvette was 1 ml. The reaction was started by adding 20 µl of the supernatant (final reaction volume 1.02 ml). All samples were analyzed in triplicate under substrate saturating conditions. The decrease in absorption was measured photometrically at 340 nm wavelength and 20°C every 18 seconds for a period of 3 minutes.

Ontogenetic differences in enzyme activity between adult and copepodite stages as well as depth-related differences in enzyme activity were statistically evaluated by Mann-Whitney U tests and Kruskall-Wallis tests, followed by a Dunn’s multiple comparison test, respectively.

## Results

### Hydrographical Parameters

**Figure 3:**
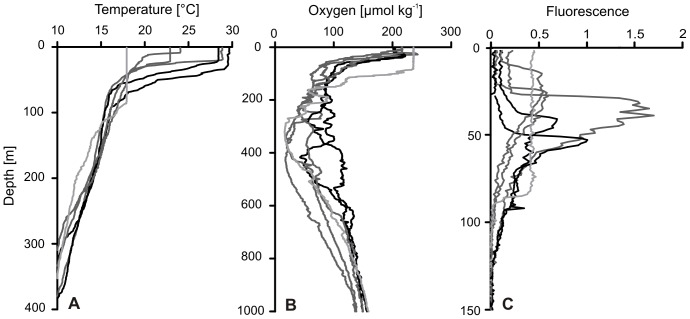
CTD profiles from stations analysed for copepod abundance. A, temperature. B, dissolved oxygen concentration. C, fluorescence (in relative units as proxy for chlorophyll *a* concentration). Shades of gray correspond to different station groups (SG1, 3, and 4) according to species composition derived from cluster analysis; SG1 = black, SG3 = dark gray, SG4 = light gray. CTD data for SG2 were not available. Note the different scaling of the y-axes.

Sea surface temperature (SST) ranged from 17.8 to 29.6°C (Table 1, Figure 3A). SST was highest at stns. 318 and 319 near the equator (SG1, black curves) and at stn. 317 (SG3, dark gray curves), while the lowest SST was recorded at stn. 840 (SG4, light gray curve) in the southern Angola Gyre. The depth of the mixed layer extended from 10 m at stn. 784 in the northeastern Angola Gyre to 86 m at stn. 840. Differences between temperature profiles were most pronounced within the upper few hundred meters, therefore, temperature profiles are only plotted down to 400 m depth.

The oxygen concentrations in surface waters exceeded 200 µmol kg^−1^ at all stations, while the depth of the upper oxycline differed greatly between stations (Figure 3B). The vertical extent and intensity of the oxygen minimum zone (OMZ, O_2_ concentrations ≤45 µmol kg^−1^) was variable and increased from north to south (Table 1). At the equatorial stns. 318 and 319 (SG1, black curves) oxygen concentrations remained above 42 µmol kg^−1^, indicating an only weakly developed OMZ. At stns. 784, 787 and 317 in the northeastern Angola Gyre (SG3, dark gray curves), lower minimum O_2_ values of 17.6 to 39.8 µmol kg^−1^ were determined between 240 and 430 m depth. In the southeastern Angola Gyre, the OMZ broadened and extended from about 50 to 400 m, with an overall O_2_ minimum of 12.7 µmol kg^−1^ (Table 1). At stn. 840 (SG4, light gray curve), oxygen concentrations of 16.4 µmol kg^−1^ were measured at 324 m. Below 400 to 600 m, O_2_ concentrations increased again above hypoxic levels.

Fluorescence, measured as proxy for chlorophyll *a* concentration, was highly variable at the different stations (Figure 3C). Lowest surface values (<0.2 in relative units) were detected at the northern tropical stns. 318 and 319 (SG1, black curves) and at the northeastern stns. 784, 787 and 317 (SG3, dark gray curves), while maximum values of >1.5 occurred in the subsurface layer (25 to 60 m) at stn. 317 (SG3). At the surface of stn. 840 (SG4, light gray curve), fluorescence was moderate (ca. 0.4 in relative units) and stayed constant down to 80 m.

### Characteristics of Station Groups Revealed by Cluster Analysis

**Figure 4:**
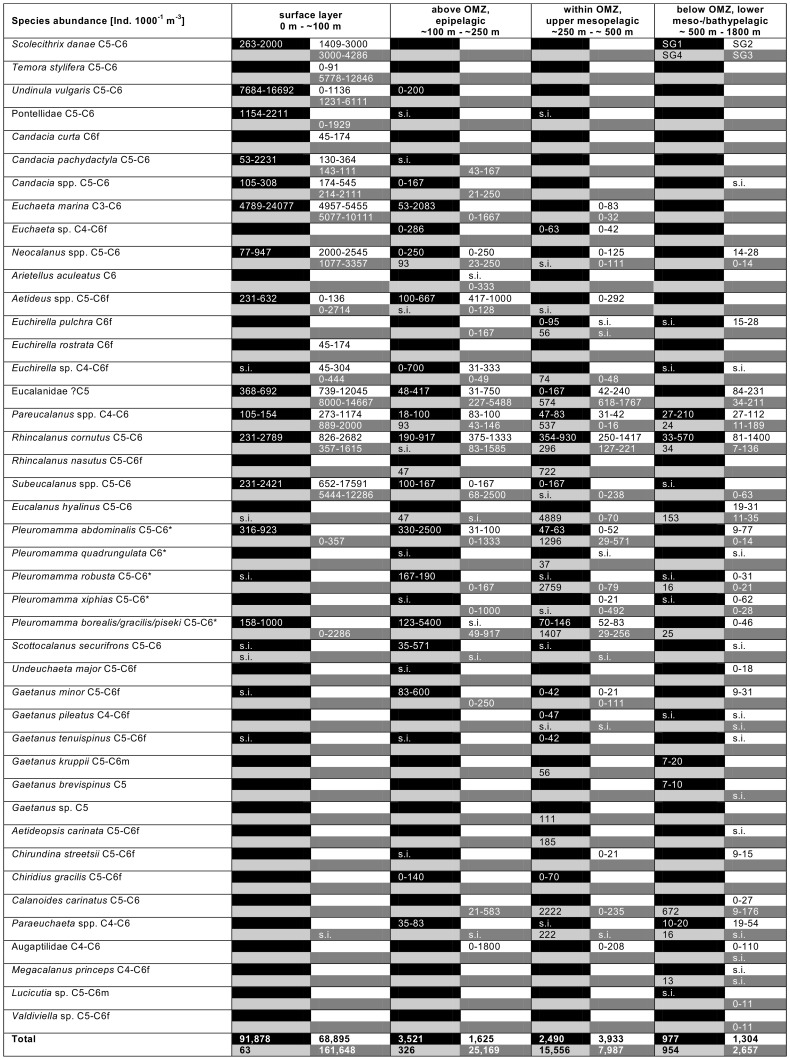
Abundance and vertical distribution of calanoid copepods in the eastern tropical Atlantic. Copepod abundance is presented for four different station groups (SG1–4) and for four different depth layers related to the vertical extent of the OMZ. Four cells per species and depth layer represent its abundance in each station group in clockwise order: top left = SG1 (black), top right = SG2 (white), bottom right = SG3 (dark gray), bottom left = SG4 (light gray), as depicted in the top right corner. * = species identified as diel vertical migrants. C3–C5 = copepodid 3–5, C6 = adult, f = female, m = male, s.i. = single individual, OMZ = oxygen minimum zone. Blank cells indicate absence.

Cluster analysis grouped the eight stations according to their similarity in copepod abundance and species composition into four station groups (SG1 to SG4, Figure 2), which is also reflected by geographic location and oceanographic features (see also Figure 1, Table 1). SG1 included the two northern equatorial stations (stns. 318, 319) with highest SST (>28°C) and only a weak OMZ (O_2_≥42.6 µmol kg^−1^). SG2 comprised the two stations located in the central Angola Basin (stns. 1, 2). The tropical stations of SG1 and SG2 were similar in total copepod abundance, but biodiversity was higher in the deep layer of SG2 (Figure 4). Stations grouped into SG3 were all located closer to the coast in the northeastern Angola Gyre (stns. 784, 787, 317) and had intermediate SST (23–29°C) and a moderate OMZ (17.6–39.8 µmol kg^−1^). Total copepod abundance was highest in almost every depth layer of SG3 compared to the other station groups (Figure 4). SG4 consisted of only one station (stn. 840) located in the southern Angola Gyre near the Angola-Benguela front and had lowest SST (18°C) and lowest O_2_ concentrations in the OMZ (16.4 µmol kg^−1^). SG4 was different from all other stations due to the lowest copepod abundance in every depth layer except within the OMZ, where copepod abundance was highest of all stations (Figure 4).

### Copepod Abundance and Distribution

In general, copepod abundance decreased with increasing depth. Abundances in the surface layer ranged from 69,000 in SG2 to >160,000 ind. 1000^−1^ m^−3^ in SG3 and decreased to about 1,000 in SG1 to 2,700 ind. 1000^−1^ m^−3^ in SG3 below 500 m depth (Figure 4). In the central Angola Basin (SG2), the copepod abundance of 3,900 ind. 1000^−1^ m^−3^ in the upper mesopelagic zone (250 to 500 m depth) was two to threefold higher than those in the adjacent depth layers above and below. Stn. 840 (SG4) close to the Angola-Benguela front showed a completely different and very peculiar vertical distribution. In the upper 250 m, copepod abundance was extremely low with 63 to 326 ind. 1000^−1^ m^−3^, whereas maximum abundance occurred in the upper mesopelagic with >15,500 ind. 1000^−1^ m^−3^. Regionally, copepod abundance was usually highest at stns. 784, 787 and 317 in the northeastern Angola Gyre (SG3). The highest biodiversity of copepods in the study area was recorded in the deepest layer of SG2 in the central Angola Basin.

Among the larger calanoid copepods, the most abundant species in the epipelagic was *Euchaeta marina* (up to 24,077 ind. 1000^−1^ m^−3^), followed by *Undinula vulgaris* and different eucalanid species (Figure 4). Eucalanidae were distributed throughout the water column and together with *Pleuromamma* spp. clearly dominated within the OMZ. Comparisons between day and night stations showed that *Pleuromamma* spp. conducted diel vertical migrations, with elevated abundance (up to 5,400 ind. 1000^−1^ m^−3^) in epipelagic waters at night (stns. 317 (SG3) and 318, 319 (SG1)) and higher abundance (up to 2,795 ind. 1000^−1^ m^−3^) within the centre of the OMZ during daytime (remaining stations). Species that exclusively occurred within the epipelagic layer were *Scolecithrix danae*, *Labidocera* spp., *Temora stylifera* and *U. vulgaris*. *Aetideus* spp., *Candacia* spp., *E. marina* and *Neocalanus* spp. were predominantly distributed at the surface, but also occurred in mesopelagic waters. *Euchirella* spp., *Gaetanus* spp., *Eucalanus hyalinus*, *Paraeuchaeta* spp. were principally distributed in the mesopelagial including the OMZ, while *Aetideopsis carinata, Lucicutia* sp., *Megacalanus princeps* and *Valdiviella* sp. mainly occurred at lower meso- to bathypelagic depths. *Calanoides carinatus* showed a bimodal distribution pattern at some stations (SG3); adults were found in epipelagic layers above the OMZ, while copepodids C5 exclusively dwelled below or within the OMZ.

The abundances of most of the typically epipelagic species were higher in the northeastern Angola Basin (SG3) compared to the central Angola Basin and equatorial area (SG1 and SG2). However, *U. vulgaris* and *E. marina* had their peak abundance at SG1 close to the equator with up to 16,692 ind. 1000^−1^ m^−3^ and 24,077 ind. 1000^−1^ m^−3^, respectively (Figure 4). Species of the families Eucalanidae (*Pareucalanus, Subeucalanus, Eucalanus* spp., *Rhincalanus cornutus*) and Metridinidae (*Pleuromamma* spp.) were distributed throughout the sampling area, except for *Rhincalanus nasutus,* which occurred only in the south (SG4). Highest abundance of eucalanids (up to 17,591 ind. 1000^−1^ m^−3^) was recorded in the epipelagic at SG2 and SG3, while metridinid species were most abundant in the equatorial region at SG1 (up to 5,400 ind. 1000^−1^ m^−3^).

The following species only occurred as single individuals at some stations and do not appear in Figure 4: *Euchirella splendens* below the OMZ at SG2; *Arietellus plumifer* above and within the OMZ at SG1; *Nannocalanus minor* above the OMZ at SG1; *Scaphocalanus magnus* above the OMZ at SG3, within the OMZ at SG1 and below the OMZ at SG2; *Gaussia princeps* above the OMZ at SG3; *Gaetanus tenuispinus* within the OMZ at SG4; *Lophothrix* sp. below the OMZ at SG2 and *Pseudochirella* sp. above and within the OMZ at SG1.

### Respiration Rates

**Figure 5:**
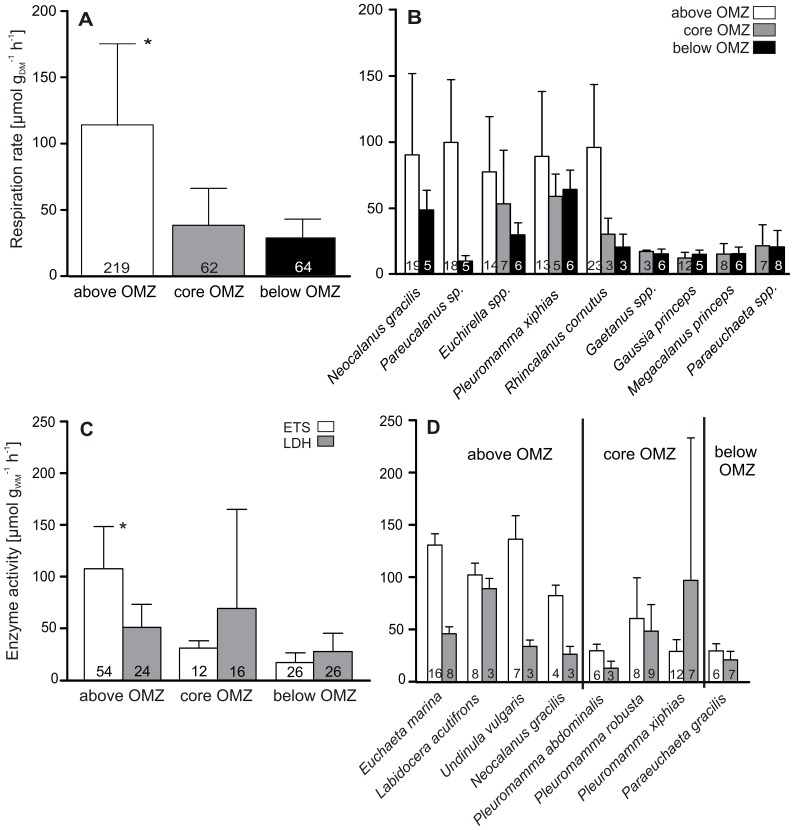
Metabolic rates of calanoid copepods in relation to the oxygen minimum zone (OMZ). A, mass-specific respiration rates. B, comparison of mass-specific respiration rates of nine copepod species (copepodids 5+adults) from different depths. C, enzyme activities of the electron transport system (ETS) and the lactate dehydrogenase (LDH). D, comparison of enzyme activities of eight copepod species (copepodids 5+adults) from different depths. Bars represent means with standard deviation: the number of measurements (n) is indicated in each bar. * = significantly higher respiration rate and ETS activity above the OMZ as compared to the core and below the OMZ.

A total of 342 individual respiration measurements were conducted with 40 calanoid copepod species. Respiration rates of copepods with ≥2 replicate measurements are shown in Table 2. Individual respiration rates varied between 2.1±0.9 µmol g_DM_^−1^ h^−1^ in copepodids C5 of *C. carinatus* from below 1000 m and 206.9 µmol g_DM_^−1^ h^−1^ in female *G. princeps* from 200–100 m depth. Mass-specific respiration ranged from 4 µmol g_DM_^−1^ h^−1^ in female *Valdiviella oligarthra* from 1800–1000 m to 273.9 µmol g_DM_^−1^ h^−1^ in male *T. stylifera* from the surface. Mass-specific respiration was significantly higher at the surface (Kruskall-Wallis test, p<0.0001) and decreased with increasing depth (Figure 5A, B). Minimum respiration rates were measured in copepods in deeper water layers below the OMZ. Figure 5B compares mass-specific respiration rates of nine copepod species from different depths above, within and below the OMZ. Mass-specific respiration rates were considerably higher in species from the surface, while there were hardly any differences in respiration between the OMZ core and below the OMZ. *Pleuromamma xiphias* and *G. princeps* had slightly lower respiration rates within the OMZ than below, but differences were not significant. The eucalanid copepods *Pareucalanus* sp. and *R. cornutus* showed considerably lower respiration in deeper waters compared to the surface. For the comparison of respiration rates between species, measurements of all stages were grouped, since differences were not significant (Mann-Whitney U test, p<0.05).

**Table 2:** Individual and mass-specific respiration rates (mean ± SD for n≥3 or range, if n = 2) of calanoid copepods from the tropical Atlantic.

Species	Stage	Samplingdepth [m]	*in situ* T.[°C]	DM range[mg]	Ind. R [nmol h^−1^] mean ± SD	DM-spec. R [µmol g_DM_^−1^h^−1^] mean ± SD	n
*Scolecithrix danae*	f	200-0	15–17.5	0.16–0.26	22.8±6.2	112.2±25.6	7
	f	50-0	18–20	0.16–0.22	32.9±10.7	169.5±49.2	6
	m	50-0	18–20	0.16/0.17	17.6/17.4	111.2/103.7	2
*Temora stylifera*	f	40-0	18–20	0.05–0.08	14.7±3.9	243.2±91.2	3
	m	40-20	18-20	0.03/0.06	8.8/3.1	273.9/51.3	2
*Labidocera acutifrons*	f	50-0	20	0.49–0.53	53.0±11.1	104.6±25.0	4
	m	50-0	20	0.48–0.54	57.0±8.6	111.4±12.3	4
*Undinula vulgaris*	f	50-0	15	0.17–0.20	31.5±8.5	174.9±59.9	4
	f	50-0	18.5–20	0.13–0.26	32.4±10.0	162.2±28.3	7
	m	50-0	15	0.18–0.20	27.9±7.7	144.0±34.7	4
	m	50-0	20	0.13/0.13	6.9/21.5	54.4/161.6	2
*Candacia bipinnata*	f	80-0	18–20	0.19–0.24	27.1±4.2	126.5±10.8	5
*Candacia curta*	f	50-0	20	0.16–0.20	15.4±4.7	83.6±21.9	3
*Candacia pachydactyla*	f	80-0	19–20	0.27/0.37	71.9/23.7	263.3/64.1	2
*Euchaeta marina*	f	220-0	13–16	0.14–0.32	18.3±10.6	86.9±64.9	6
	f	80-0	18–20	0.27–0.41	46.4±10.9	153.3±49.5	8
	f (ripe)	20-0	13	0.29–0.30	8.2±4.5	27.9±15.4	3
	f (ripe)	40-0	18–20	0.26–0.37	62.5±21.3	199.4±55.3	4
	f (eggs)	20-0	13	0.21/0.21	2.7/4.0	13.2/18.9	2
	f (eggs)	80-0	16–19	0.23–0.45	57.1±33.0	191.5±98.5	4
	m	80-0	14–16	0.23–0.25	21.7±6.7	89.7±25.3	3
	m	80-0	18–20	0.19–0.26	34.0±9.0	149.2±53.0	4
*Neocalanus gracilis*	f	800–500	7.5–8.2	0.27–0.44	19.4±6.3	50.7±11.6	10
	f	220-0	13.7–16	0.41–0.51	31.3±10.1	69.9±23.8	9
	f	80-0	18–19	0.32–0.43	61.5±30.1	161.3±86.0	5
*Neocalanus robustior*	C5	80-0	16	0.27/0.32	22.7/20.9	84.5/64.6	2
	f	220-0	14	0.51–0.63	32.6±5.7	59.3±4.5	3
	f	80-0	16–19	0.44–0.60	66.4±25.8	123.7±43.0	9
*Neocalanus* sp.	C5	100-0	14–16	0.23–0.37	15.9±4.3	58.1±9.4	3
*Arietellus aculeatus*	f	100-50	20	0.69/0.75	28.2/26.5	41.0/35.5	2
	m	100-50	20	0.69/0.79	16.4/25.0	23.6/31.6	2
*Aetideus* sp.	f	120-80	16	0.05/0.07	9.6/9.9	179.7/136.2	2
*Euchirella pulchra*	f	800-220	6.5–7.5	0.40–0.53	12.6±3.5	29.0±10.7	4
	f	500-30	10	0.40/0.52	13.6/16.0	34.0/30.8	2
*Euchirella rostrata*	f	600-50	17	0.38/0.47	45.6/49.9	119.9/105.7	2
	f	80-0	19–19.5	0.36/0.41	62.3/38.8	173.6/94.3	2
*Euchirella splendens*	f	800-40	5.5–8	0.77–1.27	27.8±7.2	28.2±7.1	5
*Euchirella* sp.	C4	80-0	16–18.5	0.20/0.24	10.6/37.3	52.4/152.9	2
	C5	800-30	7.5–9.6	0.21/0.79	5.6/21.9	26.1/27.9	2
	C5	50-100	14	0.66/0.71	57.2/51.9	86.3/73.7	2
	f	80-0	14–18.8	1.16/1.16	61.8/66.8	53.2/57.5	2
*Pareucalanus* spp.	C5	1800-1000	5–7.3	0.66–0.72	6.8±3.0	10.0±4.1	5
	C5	80-0	18.5	0.05/0.25	5.4/27.3	108.2/108.3	2
	f	220-0	13.7–16	0.17–0.41	10.3±6.7	40.0±31.8	4
	f	80-0	18–20	0.28–0.53	46.5±15.8	119.4±47.0	11
	m	50-0	18	0.65/1.08	46.6/66.8	72.2/61.9	2
*Rhincalanus cornutus*	C5	1000-200	6.5–7.5	0.09–0.20	2.8±1.1	22.9±11.5	4
	C5	220-0	14–16	0.04–0.22	10.0±3.9	83.0±36.0	8
	C5	50-0	20	0.11–0.17	17.6±4.0	129.6±30.4	5
	f	50-0	15	0.12–0.26	13.4±4.9	69.6±20.2	4
	f	50-0	18.5–20	0.15–0.26	18.5±9.8	95.6±70.8	7
*Pleuromamma abdominalis*	f	500-30	10	0.28–0.37	26.5±4.9	80.4±8.4	4
*Pleuromamma quadrungulata*	f	600-30	7.5–9.5	0.25–0.54	17.3±3.6	43.6±7.9	6
*Pleuromamma robusta*	f	100-50	15	0.25–0.34	33.3±5.4	107.3±11.1	7
*Pleuromamma xiphias*	f	1000-30	7.5–10	0.52–0.91	44.4±8.5	64.8±13.2	10
	f	200-100	14	0.35–0.61	26.2±19.9	53.2±34.9	4
	f	100-50	20	0.40–0.62	56.1±23.9	110.3±30.7	4
	m	1000-150	8–10	0.61/1.24	22.5/57.9	36.6/46.6	2
	m	100-50	20	0.46–1.10	83.7±19.5	137.2±55.2	3
*Gaussia princeps*	C5	1000-150	8	2.39–6.64	46.2±19.9	11.6±4.3	9
	f	800-200	8	8.54/9.54	103.6/101.2	12.1/10.6	2
	m	800-150	8	5.27–7.02	98.5±21.9	15.9±2.9	6
*Calanoides carinatus*	C5	1800-1000	4.7–6.2	0.11–0.14	2.1±0.9	17.0±8.7	6
	C5	400-300	10	0.10/0.11	6.6/7.4	64.4/64.9	2
*Chirundina streetsii*	f	800-400	6.5–8	1.38/1.46	36.6/26.7	26.6/18.3	2
*Gaetanus pileatus*	f	1000-250	8–10	1.02–1.72	19.7±2.7	15.0±4.3	3
*Paraeuchaeta gracilis*	f	600-400	8	1.41–2.16	41.5±29.7	23.9±18.0	4
*Paraeuchaeta hansenii*	f	800-400	8	5.89–5.98	71.8±25.0	12.0±4.1	4
*Paraeuchaeta* sp.	f	1800-500	5–7.5	1.25/1.42	19.5/18.7	15.6/13.2	2
*Megacalanus princeps*	C4/C5	1000-400	6.5–8	0.98–1.44	22.0±14.1	17.7±10.1	5
	f	1000-250	4.6	5.34/6.59	50.1/113.4	9.4/17.2	2
	f	1000-250	8	5.34–6.78	100.5±24.8	16.4±3.9	4
	m	600-400	8	3.81–4.56	49.0±11.6	11.5±1.9	3

f = female, m = male, C4/C5 = copepodid 4 and 5, T = temperature, DM = dry mass, Ind. R = individual respiration rate, DM-spec. R = mass-specific respiration rate, n = number of measurements.

### Enzyme Activities

Enzyme activities of the electron transport system (ETS) and lactate dehydrogenase (LDH) were measured for 18 and 20 copepod species, respectively (Table 3). ETS activities varied from 3.9 µmol g_WM_^−1^ h^−1^ in female *E. splendens* from 1800-1000 m depth to 170.0±22.8 µmol g_WM_^−1^ h^−1^ in *S. danae* females from the surface. LDH activities ranged from 10.6 µmol g_WM_^−1^ h^−1^ in female *Pareucalanus* sp. from 200-50 m to 375.8 µmol g_WM_^−1^ h^−1^ in *P. xiphias* males from the OMZ in 500-150 m. ETS activity was significantly higher at the surface (Kruskall-Wallis test, p<0.0001) and declined with increasing depth (Figure 5C). Differences in mean LDH activity in relation to the OMZ were not significant, although maximum LDH activities were recorded within the core of the OMZ (Figure 5C). *S. danae*, *E. marina*, *L. acutifrons* and *U. vulgaris* were among the epipelagic species with the highest ETS activities (Table 3, Figure 5D). *S. danae, L. acutifrons* and *E. marina* as well as *Candacia pachydactyla* and *R. cornutus* from above the OMZ had also high LDH activities (Table 3, Figure 5D). The overall maximum LDH activity (375.8 µmol g_WM_^−1^ h^−1^) was observed in *P. xiphias* within the OMZ. Moderate LDH activities were measured in *Pleuromamma robusta* and *Paraeuchaeta aequatorialis* from below the OMZ. For inter-specific comparison of ETS and LDH activities, measurements of all stages of one species were grouped, since differences were not significant (Mann-Whitney U test, p<0.05).

**Table 3:** Enzyme activities of the electron transport system (ETS) and lactate dehydrogenase (LDH) of calanoid copepods from the tropical Atlantic.

		ETS activities				LDH activities		
Species	Stage	Depth [m]	T. [°C]	WM range [mg]	ETS activity [µmolg_WM_^−1^ h^−1^] ± SD (n)	Depth [m]	WM range [mg]	LDH activity [µmolg_WM_^−1^ h^−1^] ± SD (n)
*Scolecithrix danae*	f	22-0	20	0.69–0.77	170.0±22.8 (3)	200-50	0.81	153.3 (1)
*Labidocera acutifrons*	f	50-0	20	2.20–2.49	100.0±8.3 (4)			
	m	50-0	20	1.98–2.14	104.4±14.7 (4)	50-0	1.97–2.27	89.1±9.7 (3)
*Undinula vulgaris*	f	50-0	20	0.77–0.88	150.2±5.0 (4)	50-0	0.80–0.84	33.9±6.0 (3)
	m	50-0	20	0.44–0.60	118.2±24.5 (3)			
*Candacia pachydactyla*	f	30-0	23	0.95–1.41	97.0±38.8 (4)	50-0	1.35/1.42	46.1/91.9 (2)
*Euchaeta marina*	C5	22-0	20	0.73–0.97	121.8±10.3 (3)			
	f	22-0	20	1.35–1.50	127.4±8.9 (4)	50-0	1.47/1.75	32.0/40.2 (2)
	f(ripe)	50-0	28	0.77–0.93	133.1±7.3 (5)	50-0	0.89–1.50	49.1±1.3 (3)
	f(eggs)	50-0	28	0.85–1.05	137.5±14.5 (4)	50-0	0.90–1.19	49.5±1.9 (3)
*Neocalanus gracilis*	f	300-100	15	1.80–2.06	82.4±10.0 (4)	300-100	1.95–2.10	26.4±7.5 (3)
*Rhincalanus cornutus*	f					250-50	0.76/1.02	20.1/49.8 (2)
*Pareucalanus* spp.	C5					1000-500	1.83–2.02	31.4±11.2 (3)
	f					1000-500	2.15–2.95	23.6±5.1 (3)
	f					200-50	2.46/3.42	16.5/10.6 (2)
*Pleuromamma abdominalis*	f	450-200	10	1.42–1.58	31.0±6.5 (4)	600-250	1.58–1.98	13.0±6.6 (3)
	m	450-200	10	1.00/2.10	22.9/31.8 (2)			
*Pleuromamma robusta*	f	100-50	15	1.40–1.50	96.7±7.3 (4)	100-50	1.38–1.39	66.4±4.6 (3)
	f	600-400	8	1.25–1.44	24.4±4.1 (4)	600-400	1.30–1.51	42.7±33.2 (4)
	f					450-200	1.62/1.68	22.3/43.7 (2)
*Pleuromamma xiphias*	f	100-50	20	2.64–3.47	28.0±15.3 (3)	100-50	3.72	39.1 (1)
	f	250-30	10	3.10–3.70	23.7±14.6 (3)	250-30	2.98	18.9 (1)
	f	500-150	10	2.60-3.40	35.5±8.0 (3)	500-150	3.25-3.6	21.2±7.3 (3)
	m	500-150	10	2.56-4.70	29.0±8.6 (3)	500-150	3.83/3.95	375.8/181.0 (2)
*Megacalanus princeps*	f	500-300	8	17.00/25.00	17.4/13.2 (2)	500-300	26.68/30.00	40.1/41.3 (2)
*Euchirella pulchra*	f	1000-450	5	2.41–2.64	10.3±3.8 (4)	450-200	2.46/2.67	48.4/80.2 (2)
*Euchirella rostrata*	f	200-30	14	1.88	100.7 (1)			
	f	1000-600	5	1.84	13.8 (1)			
*Euchirella splendens*	f	1800-1000	4	5.83/5.85	3.9/4.0 (2)	250-50	5.74	29.6 (1)
*Gaussia princeps*	f	200-100	14	12.40	32.6 (1)			
	f	1000-600	8	21.80	9.0 (1)	1000-600	35.00	16.5 (1)
	m	500-300	8	26.50	18.3 (1)	500-300	21.90	22.5 (1)
*Gaetanus brevicornis*	C5	500-300	8	2.81-3.20	11.1±1.3 (3)			
	f					500-300	5.67	15.3 (1)
*Gaetanus brevispinus*	C4/C5					1800-1000	0.91	17.8 (1)
	f	1800-1000	4	2.83	12.0 (1)			
*Paraeuchaeta aequatorialis*	f					1000-400	4.36	61.6 (1)
*Paraeuchaeta gracilis*	C5	600-400	8	3.86–4.17	26.4±8.2 (3)	600-400	3.50–3.84	20.1±13.3 (3)
	f	600-400	8	6.58–7.68	31.1±5.4 (3)	600-400	6.32–7.34	20.1±4 (4)
*Valdiviella* sp.	C3	1800-1000	4	4.90	6.0 (1)			

f = female, m = male, C3–C5 = copepodid 3–5, T = temperature, WM = wet mass, n = number of measurements.

## Discussion

The most intense oxygen minimum zones (OMZs) are located in the eastern tropical Pacific as well as in the Arabian Sea, where hypoxic waters extend over depth ranges of several hundred metres and oxygen concentrations frequently drop below 4.5 µmol kg^−1^ [4], [7], [38]. Although the OMZ in the eastern tropical Atlantic is less pronounced than in other tropical regions, the Atlantic has a high potential to suffer from major losses in dissolved oxygen within the next decades [4], [5]. The large-scale station grid sampled for the present study encompasses very diverse hydrographic regimes. The northernmost stations close to the equator were characterized by a weakly developed OMZ, whereas stations in the southern Angola Basin had a more pronounced OMZ with a minimum oxygen concentration of 12.7 µmol kg^−1^ at 410 m depth. The Angola Gyre is characterized by low oxygen concentrations in its centre and is suspected to be the source of hypoxic water for the southeastern Angola Basin and Benguela region [39], [40]. A shoaling of the OMZ in the southern Angola Basin may indicate the location of the Angola Dome (at about 10°S, 8°E), which is characterized by an uplift of isotherms and low oxygen concentrations close to the surface [7], [41].

The community analysis revealed four station groups (SG1 to SG4) that differed in copepod species composition and abundance. Abundance and distribution of calanoid copepods are comparable to other studies from the tropical Atlantic [28], [29], [42]. Calanoid copepod abundance was highest in the northeastern Angola Gyre (SG3) and decreased towards tropical waters at the equator and towards the central Angola Basin (SG1, 2). High zooplankton standing stocks in the Angola Gyre and off the coasts of Gabon and Congo have earlier been observed [43], [44]. Maximum productivity occurred in August off Gabon [43], corresponding to the time of sampling at the northern stations of SG3. The uplift of the thermocline into the euphotic zone in the area of the Angola Dome increases nutrient supply and productivity in comparison to the surrounding oligotrophic tropical waters [41]. This is also evident in the shallower mixed layer depth and subsurface fluorescence maximum of SG3 compared to other stations (Figure 3). In addition, the discharge of the Congo River provides an extra supply of nutrients, which supports enhanced biological productivity in the northeastern part of the study area [41], [45].

Copepod abundance was usually highest at the surface and continuously declined with increasing depth. This is in accordance with other findings from tropical oceans [42], [46]. One station (stn. 840, SG4) deviated from all others and contained extremely few copepods in the surface layer, but high abundances within the OMZ. This station, located in the vicinity of the Angola-Benguela front, had a deeper mixed layer and low SST, which suggests an influence of colder waters from the Benguela Current [47]. In contrast to studies from the Benguela upwelling region, the eastern tropical Pacific and the Arabian Sea [9]–[11], [26], we did not observe minimum copepod abundances within the OMZ, as compared to depth layers above and below. Biomass of pelagic organisms within OMZs does not seem to be affected by oxygen concentrations above 10 µmol l^−1^ [48], which suggests that current O_2_ concentrations in the eastern tropical Atlantic do not yet influence copepod abundance and distribution.

The OMZ was frequently inhabited by various species of the two calanoid families Eucalanidae and Metridinidae that clearly dominated over others. These species are typical inhabitants of OMZs in the Peru and Benguela Current regions as well as in the Arabian Sea [11], [17], [49], [50]. In the Arabian Sea, differences in hypoxia tolerance were observed among different eucalanid species, i.e. *Eucalanus attenuatus* and *Rhincalanus cornutus* were less common within the OMZ, while *Eucalanus elongatus* prevailed in hypoxic layers [10]. In the tropical North Pacific, *Eucalanus inermis* conducts extended ontogenetic vertical descents into the upper and lower boundary layers of the OMZ [26]. In the present study, *R. cornutus* was distributed throughout the water column, whereas *Pareucalanus* spp. and *Subeucalanus* spp. were less abundant within the OMZ but *Eucalanus hyalinus* more abundant. Several *Pleuromamma* species were identified as vertical migrants and regularly inhabited or temporally migrated into the OMZ. Since the OMZ did not seem to prevent vertical migrations of these species, they may find refuge within the OMZ from predation and competition by less tolerant species, such as fish [27].

Pelagic organisms that frequently inhabit OMZs cope with hypoxic conditions either via an efficient removal of oxygen from surrounding waters, via a reduction of metabolic rates or via additional energy from anaerobic metabolism [22]. In order to evaluate different metabolic strategies of calanoid copepods, we measured respiration rates as well as enzyme activities of the electron transport system (ETS) as a proxy for the potential aerobic rate [34], [51], and lactate dehydrogenase (LDH) as an indicator of anaerobic (glycolytic) metabolism [20]. Copepod aerobic respiration, in terms of actual respiration rate and ETS activity, was comparable to previous measurements of copepods from tropical and subtropical regions [25], [36], [52]–[54]. Mass-specific respiration rates and ETS activities were highest in copepods from surface waters and decreased with increasing depth, consistent with a decline in temperature and an increase in body mass [24], [55], [56]. While there was a rapid decline in aerobic activity below the oxygen-saturated epipelagic layer, the differences between the core of the OMZ and below were not as pronounced. Similar results have been reported from the eastern tropical Pacific [51] and Indo-Pacific [53].

A reduction of metabolic rates has been observed in organisms that inhabit OMZ regions and may be an advantage to survive within hypoxic zones [16], [19], [22], [57], [58]. Copepods of the family Eucalanidae seem to be particularly successful inhabitants of OMZs. They are often characterized by substantially reduced respiration rates and some species, such as *Rhincalanus nasutus* and *Eucalanus* spp., apparently enter a dormant state within or below the OMZ [25], [52], [54], [59]. This could also be the case for *Rhincalanus cornutus* and *Pareucalanus* sp. investigated in this study, since specimens from deeper waters showed considerably reduced locomotory activity and metabolic rates. Certain physiological traits of the “lethargic lifestyle” of eucalanid copepods have been compared to the typical adaptive strategies of jellyfish [54], which allow eucalanid species to reduce their energy expenditure and permanently colonize waters of extreme hypoxia.

In contrast, the other successful group of OMZ colonizers includes the vertically migrating copepod *Pleuromamma xiphias*. This species migrates into the OMZ twice per day and is challenged by hypoxic conditions only for limited periods of time. Similarly, *Pleuromamma* spp. and the euphausiid *Euphausia mucronata* vertically migrate through or into the OMZ in the eastern Pacific [21], [49], [60]. In *E. mucronata*, this ability correlates with an increased activity of lactate dehydrogenase [21]. In the present study, *P. xiphias* showed the highest LDH activity of all copepods, which apparently is a successful strategy to migrate into hypoxic layers. Anaerobic metabolism may thus provide additional energy for a temporal stay in the OMZ [21]–[23], while the oxygen debt can be compensated during times spent in the oxygen-rich surface layer [15], [22], [26], [61].

Increased LDH activity in surface species, e. g. *Scolecithrix danae*, *Euchaeta marina* and *Labidocera acutifrons* from this study, serves as an additional energy supply for enhanced locomotion and constant swimming [23]. Moreover, it may support the ‘sit-and-wait’ feeding strategy and burst swimming tactics typical of meso- to bathypelagic copepods such as *Paraeuchaeta aequatorialis* that also showed increased anaerobic activity [23], [62]. In general, LDH activities of tropical Atlantic copepods from the present study are lower than those of copepods off California [23]. These authors measured maximum LDH activities of 70 µmol g_WM_^−1^ min^−1^ (4200 µmol g_WM_^−1^ h^−1^), in contrast to our maximum value of 376 µmol g_WM_^−1^ h^−1^. High LDH activities have also been recorded in bulk zooplankton of the pronounced OMZ in the Peru upwelling region [63]. These differences may indicate that animals from more intense OMZs, as in the subtropical Pacific, rely to a greater extent on an anaerobic energy supply [22].

In this study, we identified different copepod communities in the eastern tropical Atlantic Ocean in relation to hydrographic regimes and, in particular, the extent of the OMZ. While calanoid copepod abundance was highest in the northeastern Angola Gyre, maximum species diversity was found in the deep Angola Basin. Copepod abundance generally decreased with increasing depth, but was not drastically reduced within the OMZ. The OMZ harboured specifically adapted species including eucalanid copepods that had a substantially reduced aerobic metabolism to permanently live within hypoxic zones, as well as diel vertical migrants such as *P. xiphias*, which showed elevated lactate dehydrogenase activity to temporally cope with hypoxic conditions during migration through the OMZ. A detailed understanding of vertical distribution patterns and ecophysiological characteristics of tropical copepods is essential to identify possible changes in the zooplankton community structure caused by expanding OMZs in the eastern tropical Atlantic.
